# An Inquiry into the Propagation of Contagious Poisons by the Atmosphere, &c. &c. &c.

**Published:** 1839

**Authors:** 


An Inquiry into the Propagation of Contagious Poisons by the Atmosphere, &c. &c. &c. By *S. Scott Alison*, M.D. Octavo. Maclachlan & Co. 1839.

This volume is written in a popular style, and designed for general perusal; but it is well worthy of the medical practitioner's attention. Would that the doctrine of contagion and infection could be divested of its imaginary terrors, and shewn in its true light to the world at large! How many thousands would then have been saved from death, and how many millions from terrors of the most dolorous kind, during the cholera-phobia of 1832-3-4, when the quarantines, purifications, and fumigations were frightening the isle from its propriety!

"The patient laid on the bed of sickness, having many wants and occasions for a thousand little offices, but being unable to assist himself, generally desires, and, where apprehension does not cause desertion, obtains the aid of good and gentle friends, whose very presence affords a gratification to the sufferer which none can sufficiently value, who have not, like him, felt its blessings. Their assistance and constant presence is absolutely necessary to supply his several wants, and to render a situation, often painful, and ever irksome, less acutely so.

But not more necessary is such assistance to the mitigation of the sufferings of the body, and the soothing, the calming of a fevered mind, than is it urgently wished for, and longed for by the patient, to whom even the momentary absence of the ministering being from his bedside is frequently the cause of much mental agitation and of pain.

But where, as we have often seen, the patient has still his senses left, and dearly loves the objects around him, what must be the amount of that bitterness of mental struggle going on in his breast, alternately heaving with desire for their presence as his greatest comfort, and with the alarm every amiable being must feel, lest those most dear to him should fall the victims of their tenderness, and be cut down themselves, in their holy endeavours to relieve his sufferings?

The apprehensions of the patient lest those kind and beloved friends ministering to his wants, and nobly incurring on his account all the risk of a dangerous situation, should unhappily derive from him, through the medium of Atmospheric Contagion, the same disease,—are calculated to produce a state of excitement highly injurious and directly opposed to that calm and cheerful state of mind so favourable to his recovery. But these apprehensions are often changed for the dreadful reality, and no little mental suffering has been produced, and no trifling obstacle to the convalescence of a patient has been raised up, by the intimation that a dear friend has caught the pestilence from him, and has in consequence been deprived of life.

The belief in the doctrine of Atmospheric Contagion is hurtful also to the friends and attendants of the patient—by its naturally conveying the impression that he is a centre of a poisonous agent, whose immediate tendency is to propagate the distemper and diffuse itself through the atmosphere, extending to it, its deleterious attributes, to be felt by all who respire it." 10.

In another chapter our author goes, we fear, a step too far—namely, in coming to the conclusion that "atmospheric contagion has no existence, properly considered, in the light of an atmosphere holding in solution a specific contagious poison." He admits, however, that contagion, such as that of small-pox or syphilis, may be communicated by contact, or by the impregnation of fomites. But he questions the atmospheric contagion, for these reasons:—

"*First*, That in the whole course of its history, it fails to supply us with sufficient evidence thereof; *secondly*, That its supposed career is not marked with the same uniformity of effect, and constancy of character, cognisable among other powerful agents, but appears rather to be regulated by no fixed laws; *thirdly*, That the phenomena of disease do not go to shew that it is dependent on atmospheric contagion, the occurrence and dissemination of which, moreover, it could not explain.

We are further disposed to deny its existence at all, for this reason, that its admission is opposed to the testimony of direct observation and of experiments instituted for the purpose." 24.

The greater part of the work is occupied with illustrations and historical facts, supporting the above propositions. The reasoning is close and logical—and cannot be analyzed. The whole must be read with attention, and is well calculated to effect good purposes.

It is a general rule that in epidemic diseases, those who have or have had communication with the sick, are not more prone to the malady than others who have not had such communication. But our author has judiciously admitted some exceptions. These are :—

1st. The relations and inmates of the same house inhabited by one sick of fever.

2d. Those receiving disease from actual contact with the palpable contagious matter, or by contactual contagion.

3d. Those persons, through the operation of fear, and from depression of mind, affected with disease, as fever, cholera, &c.

4th. The attendants in fever institutions, &c." 71.

Every intelligent person must readily comprehend how these exceptions may be explained without admitting the transmission of specific virus from one individual to another. It is, however, almost entirely on these exceptions that the contagionists depend for the maintenance of their gloomy doctrines. On this account it will probably be prudent to dwell a little on these apparent exceptions.

In respect to the *first*, it is hardly necessary to observe that the relatives of patients, if inhabiting the same localities, must be as liable to sickness as the sick themselves. They are generally suffering under depression, and fear of infection—they are irregular in their meals and rest, having little appetite, and harassed by night-watching and other debilitating agents. They also respire an impure atmosphere. In fine, the wonder is that so many friends and attendants escape disease under such disadvantageous circumstances.

The 2nd exception we shall quote entire.

"Visitors and attendants are liable to increase their ordinary chance of taking the prevalent distemper, by touching the body or clothes of the sick, when he labours under a disease marked by a palpable contagious poison. Though the poison cannot be diffused through the air, it may, and sometimes does, act by contact, which we call contactual or immediate contagion. That, of course, can operate in those diseases only in which a palpable poison is eliminated. Those diseases are in this country chiefly small-pox, chicken-pox; the plague, if it can now be said to be a disease of this country; the itch; and, as is commonly believed, measles, scarlet fever, &c. &c.

Though the propagation of these diseases may take place from contact with their peculiar contagious matters, we are disposed to think, that, at least with most of them, especially the latter, the cases which occur in that way are very few.

It is sometimes difficult to produce disease, even when the skin is cut, and the matter is then introduced. That step sometimes fails in respect to cow-pox matter, even when fresh; and small-pox matter we have known to be in contact with the tips of the finger for a minute or so, in innumerable cases, as in feeling the pulse, and no disease has followed. Women affected with small-pox bear healthy children.

Almost the only diseases which we are disposed to think are propagated by contact with the person or clothes of the sick, are small-pox, chicken-pox, scabies, plague, &c. They all possess palpable matters in abundance.

Many instances are known to us, where children have got small-pox, and of grown-up people who have got itch, from sleeping with those sick of these distempers, and thus coming in contact with them closely, and for some time; and where they have not been seized with them, when only breathing the same atmosphere used by those sick.

Thus visitors and attendants may get disease by contact with palpable contagious matter, that is, by contactual contagion, and by touching clothes impregnated with the same, that is, by fomitic contagion, which they would not have taken, had they merely been respiring the air used by the patients." 77.

The question will here naturally occur to most minds—how do people catch the small-pox, &c. who have no communication with the sick? The answer will be—that the poison is then in the air; but not emanating from the bodies of the infected. This is rather a doubtful doctrine. If the poison can be transmitted by inoculation, it is not very unreasonable to suppose that it can be transmitted through the air of an apartment, a street, or a town. It is true that the infection of syphilis cannot be thus transmitted. It must have contact. But all poisons may not be so ponderous as that of lues. If odours are capable of being transported to considerable distances through the air, we do not see why certain morbid poisons may not be so also. Our author admits that the following fact is a very obstinate one, and not easily overcome.

"The history of the Royal Infirmary of Edinburgh, and of Queensberry House, an institution for the reception of fever patients, shews that on some occasions almost all the clerks and nurses waiting upon those affected with fever, have been seized with it also.

And it would appear to be owing to some agency peculiar to fever patients or their wards; for in regard to the first-mentioned institution, it is ascertained that it is with those attendants only, who wait upon those patients, that the greater amount of sickness is experienced. Those attendants exclusively occupied in the surgical wards being attacked in no greater proportion than those unconnected with the institution." 79.

The author exerts all his ingenuity to overcome these obstinate facts. In the first place, he observes that such occurrences do not take place in all institutions of this kind—and therefore that there may be some peculiarity about the localities not yet ascertained. He thinks it is owing to the general "*contamination of the atmosphere*," by morbid effluvia from the bodies of the sick. This will be granted by most of the contagionists—but then how comes it that the diseases are identical with those of the patients first affected? The case, however, is argued with great ability, and the whole work deserves attentive perusal. We have been able only to glance at the first two chapters, since an analysis of the whole book would require a fourth part of the journal, so closely and tersely are the arguments stated and the deductions drawn. We strongly recommend the volume to the profession.

